# Differential Assembly of Native Epithelial Sodium Channel Complexes Across Mouse Epithelial Tissues

**DOI:** 10.34067/KID.0000001261

**Published:** 2026-06-15

**Authors:** Arpita Bharadwaj, Joshua N. Curry, Xiao-Tong Su, Romina Barria Maturana, James A. McCormick, David H. Ellison, Isabelle Baconguis

**Affiliations:** 1 Vollum Institute, Oregon Health and Science University, Portland, Oregon; 2 Division of Nephrology and Hypertension, Department of Medicine, Oregon Health and Science University, Portland, Oregon; 3 Department of Chemical Physiology and Biochemistry, Oregon Health and Science University, Portland, Oregon

**Keywords:** epithelial, epithelial sodium channel, ion channel, membranous nephropathy, sodium (Na^+^) transport

## Abstract

**Key Points:**

- The new mouse line, epithelial sodium channel γ - mVenus FLAG (ENaC*γ*-VF), expresses a *γ* subunit tagged with mVenus and 3xFLAG that retains wild-type physiological function at baseline.
- The tags allow fluorescence-based detection and affinity purification of native *γ*-containing epithelial sodium channel complexes under mild, nondenaturing conditions.
- *γ*-containing complexes are heterogeneous, including *αβγ* channels as well as complexes associated with channel biogenesis, trafficking, and regulation.

**Background:**

The epithelial sodium channel (ENaC) governs sodium and fluid absorption in kidney, lung, and colon epithelia, but the molecular organization of native ENaC complexes *in vivo* remains poorly defined. Low abundance and biochemical instability have limited direct analysis of ENaC assembly, composition, and regulatory associations in native tissues.

**Methods:**

We generated a knock-in mouse in which the endogenous ENaC*γ* subunit was fused to a fluorescent protein and affinity tag, while preserving physiologic channel function. We validated channel activity *in vivo* using electrolyte measurements and pharmacologic inhibition and assessed channel pharmacology by radioligand binding. Native ENaC complexes were analyzed from lung, kidney, and colon using fluorescence-detection size-exclusion chromatography, single-molecule pull-down, fluorescent antibody fragments, and mass spectrometry.

**Results:**

The tagged *γ* subunit preserved normal ENaC function *in vivo*. Native ENaC complexes directly isolated from lung, kidney, and colon, revealed marked tissue-to-tissue differences in channel abundance and apparent complex size. Dual-color fluorescence analyses distinguished fully assembled channels from broader *γ*-containing assemblies. Proteomic analysis identified regulatory proteins associated with *γ*-containing complexes, indicating that ENaC exists in multiple assembly and regulatory states *in vivo*.

**Conclusions:**

Native ENaC architecture is heterogeneous across epithelial tissues. Endogenous tagging enables direct molecular interrogation of ENaC assembly and regulation *in vivo*.

## Visual Abstract

**Figure:**
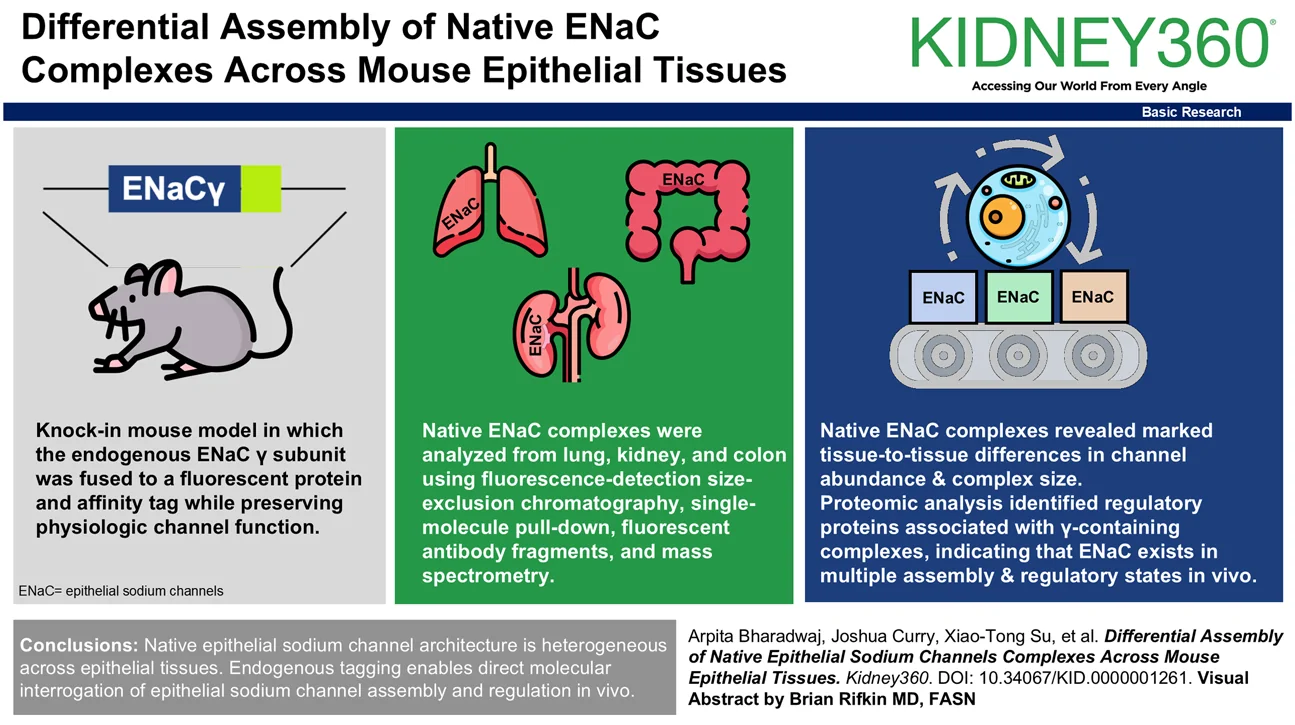

## Introduction

Transport across epithelial barriers depends on controlled sodium movement mediated by the epithelial sodium channel (ENaC). Situated at the apical membrane of epithelial cells, ENaC governs extracellular fluid balance,^1^ BP regulation,^2^ and airway surface hydration.^2–4^ Dysregulation of ENaC contributes to cystic fibrosis^5^ and pseudohypoaldosteronism,^6,7^ pulmonary edema,^8^ and hypertension.^9–20^ Despite its central physiological importance, the molecular organization of ENaC in native tissues remains poorly defined.

Recombinant expression systems have provided detailed insights into ENaC structure,^21–23^ gating,^24^ proteolytic activation,^25,26^ and pharmacologic inhibition.^27–29^ These approaches, typically involving heterologous overexpression of ENaC subunits, enable precise experimental control and mechanistic dissection of channel function. However, they may not recapitulate tissue-specific assembly pathways, potentially obscuring native regulatory mechanisms. By contrast, studies of native ENaC offer access to physiologically assembled channels operating within their endogenous regulatory and lipid environments. *In vivo*, ENaC expression and regulation vary across epithelial tissues, where differences in subunit composition, processing, and interacting partners likely give rise to distinct channel assemblies and properties that cannot be faithfully reproduced in recombinant systems.^30–32^ Native tissue approaches such as electrophysiology and transcriptomic analyses provide important information about channel function and expression; however, they do not directly reveal how ENaC subunits assemble into functional complexes or which regulatory factors shape those assemblies *in vivo*. One reason why relatively little is known about the three-dimensional information of native ENaC complexes is that it is expressed at low levels, is biochemically labile, and resides in membranes that complicate solubilization.^33–35^ To fully understand ENaC function in its physiological context, approaches are needed that enable biochemical, biophysical, and pharmacological interrogation of intact native channel complexes.

To address this longstanding bottleneck, we developed a genetically engineered knock-in mouse line, epithelial sodium channel γ - mVenus FLAG (ENaC*γ*-VF), in which the endogenous *γ* subunit is fused at its C terminus to mVenus, followed by a 3C protease site and a 3xFLAG epitope.^36–38^ This multifunctional tag enables fluorescence-based detection, affinity purification, and gentle protease-mediated elution of intact ENaC complexes, while preserving native expression levels, regulatory control, and tissue specificity. Here, we validate that the tagged *γ* subunit supports normal ENaC function *in vivo* and demonstrate that it is compatible with fluorescence-detection size-exclusion chromatography (FSEC)^39^ and single-molecule pull-down (SiMPull),^40^ permitting direct quantification of native ENaC assemblies isolated from lung, kidney, and colon. In parallel, we developed a fluorescent antigen-binding (Fab) fragment targeting ENaC*α*, enabling dual-color, subunit-resolved analysis of ENaC architecture in native lysates. These tools permit direct interrogation of ENaC architecture across epithelial tissues.

## Methods

### Generation of ENaC*γ*-VF Mice

The ENaC*γ*-VF knock-in mouse line was generated on a C57BL/6 background by the Jackson Laboratory using Clustered Regularly Interspaced Short Palindromic Repeats (CRISPR)/Cas9-mediated genome editing to insert a sequence encoding mVenus, a 3C protease cleavage site, and a 3xFLAG tag at the C-terminus of the endogenous *Scnn1g* gene. Heterozygous animals (*ENaCγ-VF/^-^*) were initially shipped to our laboratory for validation. After phenotypic and localization analysis (see Results), the Jackson Laboratory provided homozygous (*ENaCγ-VF/ENaCγ-VF*) mice, which were also viable and showed normal ENaC*γ* localization. Both heterozygous and homozygous mice are maintained under standard breeding protocols. To ensure continued integrity of the tagged allele, we routinely verify the line using genotyping services with primers designed to detect the presence of the mVenus-3xFLAG insertion. All animal procedures were conducted in accordance with Oregon Health and Science University (OHSU) Institutional Animal Care and Use Committee protocols.

### Immunofluorescence Microscopy

Mice were anesthetized with a ketamine-xylazine-acepromazine cocktail (50:5:0.5 mg/kg). Kidneys were perfusion-fixed by retrograde abdominal aortic perfusion of 3% paraformaldehyde in PBS (pH 7.4). After perfusion, the kidneys were removed, dissected, and cryopreserved in 800 mOsm/L sucrose in PBS overnight before embedding in Tissue-Tek Optimal Cutting Temperature compound (Sakura Finetek, Torrance, CA). Slides were prepared by cutting 5 mm sections and stored at −80°C until use. For immunofluorescence, sections were permeabilized with 0.5% Triton X-100 in PBS for 30 minutes and blocked with 5% milk in PBS for 30 minutes. Sections were then incubated with primary antibodies diluted in blocking buffer for 1 hour at room temperature or overnight at 4°C. Primary antibodies included FITC-conjugated goat anti-green fluorescent protein (anti-GFP; Rockland, 1:100) for mVenus detection, rabbit anti-rat ENaC*γ* (Stressmarq, 1:500), mouse anti-Aquaporin-2 (Santa Cruz, 1:50), rabbit anti-ENaC*α*^41^ (Loffing Lab, 1:500), and chicken anti-phosphorylated sodium-chloride cotransporter^42^ (Welling Lab, 1:5000). After three washes with PBS, sections were incubated with fluorescent dye-conjugated secondary antibodies diluted in blocking buffer for 1 hour at room temperature. Secondary antibodies included Alexa Fluor 488 donkey anti-goat (1:500), Alexa Fluor 555 donkey anti-rabbit (1:500, for ENaC*γ*), Alexa Fluor 555 donkey anti-mouse (1:500), Alexa Fluor 647 donkey anti-rabbit (1:500), and Cy3 donkey anti-chicken (1:500). Sections were washed with PBS three times and stained with 4′,6-diamidino-2-phenylindole (DAPI; Thermo Fisher Scientific, 1:5000), then mounted using ProLong Diamond Antifade Mountant (ThermoFisher Scientific, Carlsbad, CA). Images were acquired using a KEYENCE BZ-X800 microscope (Itasca, IL).

### Amiloride Treatment and Urine Analysis

Mice were injected intraperitoneally with vehicle (0.9% saline) and then placed in metabolic cages for a 6-hour urine collection. Five days later, the same animals received intraperitoneal injection of amiloride hydrochloride (40 *μ*g per 25 g body wt), followed by another 6-hour urine collection. Sodium and potassium concentrations were measured by flame photometry, and the urinary Na^+^: K^+^ ratio was used to assess ENaC function.

### Plasma Aldosterone Measurement

Whole blood was collected under anesthesia (isoflurane) through open cardiac puncture. Blood chemistries were measured using an i-STAT point-of-care autoanalyzer (Chem 8+, Abbott). Plasma aldosterone was measured using competitive ELISA (Immuno-Biological Laboratories America #IB79134).

### Western Blot

To compare the banding pattern of ENaC*γ*-VF and wild-type tissues, lungs, and kidneys from two mice and colons from four mice were collected, homogenized in solubilization buffer containing 50 mM tris (hydroxymethyl)aminomethane (Tris)-HCl (pH 7.6), 200 mM NaCl, 2 mM ATP, 2mM MgSO_4_, 1% digitonin (high purity, Millipore Sigma), nuclease, and protease inhibitors. Before incubation with anti-FLAG M2 magnetic beads (Millipore Sigma), 30 *µ*l of clarified lysate was saved for Western blot analysis. Identical solubilization conditions were used for wild-type tissues in parallel. To compare the banding pattern of isolated *γ*-containing samples with that of the input lysate, clarified lysates from ENaC*γ*-VF tissues were incubated with 50 *µ*l of anti-FLAG M2 magnetic beads overnight at 4°C with gentle rotation. Beads were washed ten times with 5 ml wash buffer (20 mM Tris-HCl [pH 7.6], 200 mM NaCl, 0.1% digitonin), and proteins were eluted by 12 *μ*g of PreScission Proteases (Genscript). The eluted material, in a total volume of 160 *µ*l, was used for Western blot analysis. Samples were resolved on 4%–15% Criterion Tris-HCl SDS-PAGE gels and transferred to polyvinylidene fluoride (PVDF) membranes according to the manufacturer's instructions (Bio-Rad). To detect ENaC*γ*, membranes were blocked with 5% nonfat milk in tris buffered saline (TBS; 20 mM Tris-HCl [pH 7.6], 200 mM NaCl) for 1 hour and incubated overnight with rabbit anti-rat ENaC*γ* antibody (StressMarq, 1:1000). To detect mVenus, membranes were blocked with 5% non-fat milk in TBS overnight and then incubated the following day with rabbit anti-GFP antibody (Invitrogen, 1:4000) for 2 hours. Membranes were washed with Tris-buffered saline with Tween 20 (20 mM Tris-HCl (pH 7.6), 200 mM NaCl, 0.1% tween-20) and incubated with IRDye 800CW secondary antibody (LICORbio, 1:20,000) for 1 hour before imaging.

### Filter Binding Assay

Immediately after euthanasia, kidneys were dissected from adult wild-type or ENaC*γ*-VF mice, rinsed in ice-cold TBS (20 mM Tris-HCl [pH 7.6], 200 mM NaCl), stored in TBS with protease inhibitors (Thermo Scientific Pierce protease inhibitor tablets, ThermoFisher Scientific), and flash-frozen in liquid nitrogen. For membrane preparation, frozen kidneys were pulverized using a liquid nitrogen-cooled mortar and pestle until a fine powder was obtained. Tissue was then homogenized in homogenization buffer (50 mM phosphate buffer, pH 7.5) using a Dounce homogenizer. The homogenate was centrifuged at 1800×*g* for 10 minutes at 4°C to remove nuclei and cellular debris. The resulting supernatant was further centrifuged at 100,000×*g* for 60 minutes at 4°C to pellet membrane fractions. Membrane pellets were resuspended in binding buffer (50 mM phosphate buffer, pH 7.5). Equilibrium ligand binding was assessed using tritiated benzamil ([^3^H] benzamil; Moravek). Membrane suspensions (typically 10 mg) were incubated with increasing concentrations of [^3^H] benzamil (0.5–300 nM) in a final volume of 500 *µ*l binding buffer. Incubations were performed at 22°C for 2 hours, a duration sufficient to reach equilibrium under these conditions. Nonspecific binding was determined in parallel samples containing 100 *µ*M unlabeled phenamil (Phenamil mesylate, Tocris Bioscience). Binding reactions were terminated by rapid vacuum filtration onto presoaked glass microfiber filters (Glass Fiber Filter Grade B 25 mm, Whatman) using a 12-well filtration manifold (1225 Sampling Manifold, Millipore). Filters were prewashed with 2 ml ice-cold binding buffer and treated with 0.3% polyethyleneimine (molecular weight 25K, Polysciences) to reduce nonspecific radioligand adsorption. Incubated samples were applied, and each well was washed immediately with 2×5 ml ice-cold binding buffer. Filters were dried by vacuum, transferred to scintillation vials, extracted in 5 ml scintillation cocktail (Ultima Gold, Revvity), and radioactivity was quantified by liquid scintillation counting (Beckman Coulter LS6500). Specific binding was calculated as total minus nonspecific counts per minute. Equilibrium binding curves were fitted to a one-site binding model using nonlinear regression in GraphPad Prism:

$$Y=\frac{B_{\max}\;X}{K_{d}+X}$$

where *Y* is specific binding, *X* is the free ligand concentration, *B*_max_ is maximal binding, and *K*_*d*_ is the apparent *K*_*d*_. Data from ENaC*γ*-VF and wild-type samples were fitted independently, and similar *K*_*d*_ values were interpreted as evidence that the tagged *γ* does not perturb benzamil affinity.

### Tissue Preparation and Lysate Generation for FSEC Analysis

For each FSEC experiment, one lung (all lobes), one kidney, and two colons were collected, rinsed in cold TBS, and weighed. Each tissue was homogenized in solubilization buffer containing 100 mM Tris-HCl (pH 7.6), 200 mM NaCl, 1% *n*-dodecyl-*β*-D-maltoside (DDM, Anagrade, Anatrace), protease inhibitors and Pefabloc small compound (Millipore Sigma). The homogenization volume was standardized to 750 *µ*l per 100 mg of tissue. Samples were solubilized at 4°C for 60 minutes with gentle rotation, then clarified by centrifugation at 100,000×*g* twice for 40 minutes each. Supernatants were concentrated using a 100 kDa centrifugal concentrator to a final volume of 110–480 *µ*l. Concentrated samples were then centrifuged again at 100,000×*g* for 10 minutes to remove aggregates. The final cleared supernatant was injected onto a Superose 6 Increase 10/300 glass column (Cytiva) for FSEC analysis (Waters high-performance liquid chromatography).^39^ Fractions were collected and analyzed on the basis of mVenus fluorescence.

### SiMPull Assays

Glass coverslips were cleaned and PEGylated using standard silanization procedures,^40^ then functionalized sequentially with streptavidin and a biotinylated anti-GFP/mVenus nanobody.^43^ One experimental channel included the nanobody to capture fluorescently tagged ENaC subunits, while a parallel channel without nanobody was used to quantify background signal.

Streptavidin (Invitrogen) was applied at a concentration of 250 *µ*g/ml and incubated for 5 minutes at room temperature, followed by a wash with the assay buffer (20 mM Tris-HCl [pH 7.6], 200 mM NaCl, 0.025% DDM) containing 200 *µ*g/ml BSA (Invitrogen). Biotinylated nanobody was then applied at 3 *µ*g/ml and incubated for 10 minutes, followed again by a wash to remove unbound reagent. Clarified cell lysates (prepared as for FSEC analysis) containing fluorescently tagged ENaC subunits were then applied and incubated for 10 minutes at room temperature, followed by a final wash before imaging. Chambers were imaged on a Leica Digital Microscope Inverted Series 8 TIRF microscope with a 100× oil-immersion objective. Images were captured using an Andor iXon Ultra 888 back-illuminated Electron Multiplying Charge-Coupled Device camera with a 133×133 *µ*m imaging area and 130 nm pixel size. For counting fluorophore spots, images were acquired using the excitation wavelength 488 nm. Spot detection and localization were performed using the ComDet plugin in ImageJ (Fiji). Molecule positions were analyzed within the center area of the field of view (512×512 px). The approximate particle size was set at five pixels. The spot intensity threshold (in SD) was set to three.

### Affinity Isolation of Native ENaC Complexes for FSEC and Mass Spectrometry

Tissue preparation for this assay was performed using the same protocol as described for FSEC analysis, with the exception that digitonin was used as the detergent for solubilization instead of DDM. Lungs, kidneys, and colons were collected from five mice and homogenized in solubilization buffer containing 100 mM Tris-HCl [pH 7.6], 200 mM NaCl, 5 mM EDTA, 1% digitonin (High purity, Millipore Sigma), protease inhibitors, and Pefabloc. Clarified lysates were incubated with 50 *µ*l of anti-FLAG M2 magnetic beads (Millipore Sigma) overnight at 4°C with gentle rotation. Beads were washed ten times with 15 ml wash buffer (20 mM Tris-HCl [pH 7.6], 200 mM NaCl, 0.1% digitonin), and proteins were eluted by incubating with 25 *µ*g of PreScission Proteases (GenScript) for 6 hours at 4°C. The eluted material, in a total volume of 400 *µ*l, was concentrated using centrifugal filters prior to injection onto a Superose 6 Increase 10/300 GL column (Cytiva) equilibrated in FSEC buffer (20 mM Tris-HCl [pH 7.6], 200 mM NaCl, 0.1% digitonin). Fluorescence was monitored using an inline fluorimeter (Shimadzu RF-20A) to track mVenus-tagged ENaC*γ*. Fractions corresponding to the fluorescent signal were collected, pooled, and concentrated again for downstream mass spectrometry analysis.

### 7B1-mScarlet-I Fab Production

DNA sequences encoding the heavy and light chains of the Fab fragment of the anti-ENaC*α* mAb 7B1 were cloned into separate plasmid Eric Gouax BacMam expression plasmids.^21,44^ The heavy chain construct was engineered to include mScarlet-I at the C-terminus, followed by a Strep-tag II for affinity purification.^45^ Both constructs included an *N*-terminal leader sequence (MGWSCIILFLVATATGVHS) that allows for secretion of the Fab into the media.^46^ First passage of Baculovirus was generated from each plasmid using Sf9 insect cells cultured at a density of 0.5×10^6^ cells/ml at 27°C for 5 days. P1 virus was used to reinfect Sf9 insect cells cultured at a density of 1×10^6^ cells/ml at 27°C for 4 days. After incubation, cells were pelleted by centrifugation at 1800×*g* for 20 minutes, and the supernatant containing the virus was harvested and filtered through a 0.22 *µ*m filter. The resulting virus was used to infect suspension-adapted human embryonic kidney (HEK)293S GnTI- (ATCC CRL-3022, ATCC) cells at a density of 3×10^6^ cells/ml and incubated at 37°C for 8 hours. After 8 hours, 10 mM sodium butyrate was added, and the cells were moved to a 30°C incubator. Cells were cultured at 30°C for 96 hours, after which they were spun down, and the culture supernatant was collected and filtered again using a 0.22 *µ*m filter. The clarified media with BioLock (1.6 ml/L of FreeStyle 293, IBA Lifesciences) was loaded onto a 10 ml Strep-Tactin affinity resin (5 ml/L of media) (Cytiva) pre-equilibrated with wash buffer (20 mM Tris [pH 7.6], 200 mM NaCl and 0.025% DDM). The column was washed with 20 column volumes of wash buffer, and bound 7B1-mScarlet-I Fab was eluted using 2.5 mM desthiobiotin-containing wash buffer. Desthiobiotin (IBA Lifesciences) was subsequently removed using a protein desalting-10 desalting column (Cytiva), and the Fab was eluted in wash buffer. Purity and labeling of the Fab were confirmed by SDS-PAGE and FSEC.

### Cell-Surface Biotinylation and Validation of 7B1-mScarlet-I Binding to Recombinant ENaC

To evaluate binding of the 7B1-mScarlet-I Fab to recombinant mouse ENaC, we expressed a mouse ENaC construct composed of the *α* (Uniprot ID Q61180), *β* (Uniprot ID Q9WU38), and *γ* (Uniprot ID Q9WU39) subunits, with the *γ* subunit bearing the C-terminal mVenus tag. Cells were analyzed under both total and plasma membrane-enriched conditions. For total protein analysis, cells were solubilized directly,^22,23^ clarified by centrifugation, and the resulting lysates were incubated with 7B1-mScarlet-I before downstream analysis. To specifically assess plasma membrane-expressed ENaC, cell-surface proteins were labeled using a membrane-impermeant biotinylation reagent according to the manufacturer's instructions (Pierce Cell surface biotinylation and isolation kit, ThermoFisher Scientific). Briefly, cells expressing recombinant mouse ENaC were harvested and incubated with biotin (EZ-Link Sulfo-NHS-SS-Biotin) to selectively label surface-exposed proteins. Excess biotin was quenched and removed by washing, after which cells were either flash-frozen for storage at −80°C or processed immediately for solubilization.

Cells were lysed in buffer containing 20 mM Tris-HCl (pH 7.6), 150 mM NaCl, 1% DDM, 0.18% cholesteryl hemisuccinate, and protease inhibitors. Lysates were clarified by centrifugation, and the supernatant was incubated with green fluorescent protein nanobody resin using previously described conditions.^22,23^ After binding, the resin was washed with 20 mM Tris-HCl (pH 7.6), 150 mM NaCl, 0.025% DDM, 0.0045% cholesteryl hemisuccinate without and with 5 mM CaCl_2_. Both biotinylated and non-biotinylated fractions of the protein were eluted by thrombin (30 *µ*g/ml of resin, human *α*-thrombin, Prolytix) cleavage. The thrombin eluted sample was passed through the Strep-Tactin resin to capture biotinylated, plasma membrane-expressed proteins. The unbound flow-through, representing the intracellular protein fraction, was also collected. Following fractionation, 7B1-mScarlet-I was added to the flow-through fraction. Biotinylated proteins bound to the Strep-Tactin resin were subsequently eluted using excess biotin (50 mM from 10× Buffer BXT, IBA Lifesciences), and 7B1-mScarlet-I was added to the eluted plasma membrane fraction. Both intracellular (flow-through) and plasma membrane (biotin-eluted) fractions were analyzed by high-performance liquid chromatography, with fluorescence monitored in the mScarlet channel.

### Statistical Analysis

Statistical analyses were performed using GraphPad Prism. Body weight data are presented as mean±SD and were analyzed using an unpaired two-tailed *t* test. Plasma aldosterone measurements are presented as mean±SEM and were analyzed using a two-tailed *t* test. Amiloride response experiments were analyzed using two-way ANOVA, and data are presented as mean±SEM. SiMPull data were analyzed using unpaired two-tailed *t* test. A value of *P* < 0.05 was considered statistically significant.

## Results

### Generation and Validation of the ENaC*γ*-VF Mouse Line

To enable detection and purification of native ENaC complexes, we designed a knock-in mouse line in which the endogenous *Scnn1g* locus (encoding ENaC*γ*) was modified to incorporate a C-terminal mVenus^Q69M^ fluorophore, a 3C protease recognition site, and a 3xFLAG epitope^47,48^ (Figure 1A). The tag was inserted in-frame at the endogenous *Scnn1g* stop codon using CRISPR/Cas9.^49,50^ During the initial characterization of the mouse line, confocal imaging confirmed correct apical localization of the tagged *γ* subunit in the distal nephron (Figure 1, B and C and Supplemental Figure 1).^51^ Consistent with this, both heterozygous *ENaCγ-VF/*^*-*^ and homozygous *ENaCγ-VF/ENaCγ-VF* mice were viable, exhibited normal growth, and were phenotypically indistinguishable from wild-type animals in our colony (Figure 2A). All subsequent physiological, biochemical, and biophysical analyses were therefore performed using homozygous ENaC*γ*-VF mice.

**Figure 1:**
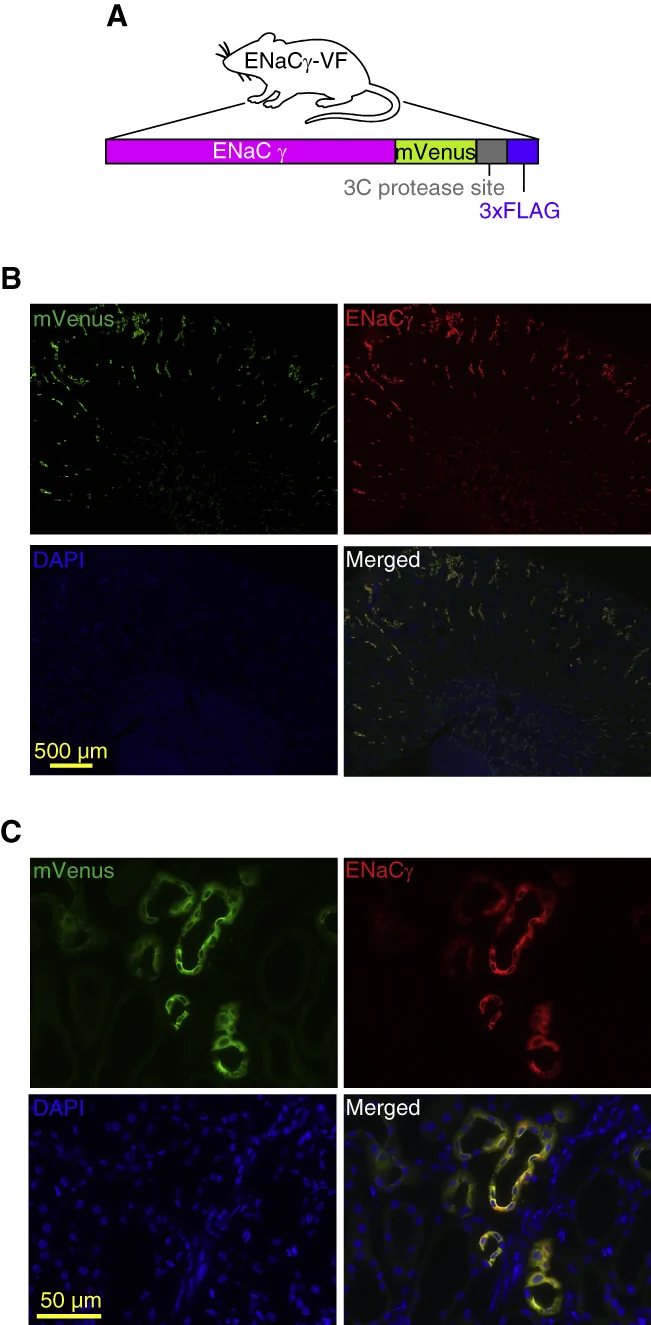
**Immunofluorescence staining of kidney tissue from homozygous ENaC*γ*-VF mice.** (A) Schematic of the tagged ENaC*γ* subunit produced from the ENaC*γ*-VF allele, which includes a C-terminal mVenus, 3C protease site, and 3xFLAG tag. (B) Representative immunofluorescence image showing colocalization of mVenus and ENaC*γ* in kidney tissue. mVenus signal was detected using a FITC-conjugated anti-GFP primary antibody (goat, Rockland, 1:100) and Alexa Fluor 488 donkey anti-goat secondary antibody. Endogenous ENaC*γ* was detected using a rabbit anti-ENaC*γ* primary antibody (StressMarq, 1:500) targeting the C-terminus, and Alexa Fluor 555 donkey anti-rabbit secondary antibody. (C) Close-up view highlighting localization of tagged ENaC*γ*. Anti-GFP, anti-green fluorescent protein; DAPI, 4′,6-diamidino-2-phenylindole; ENaC, epithelial sodium channel;  ENaC*γ*-VF, epithelial sodium channel γ - m Venus FLAG.

**Figure 2:**
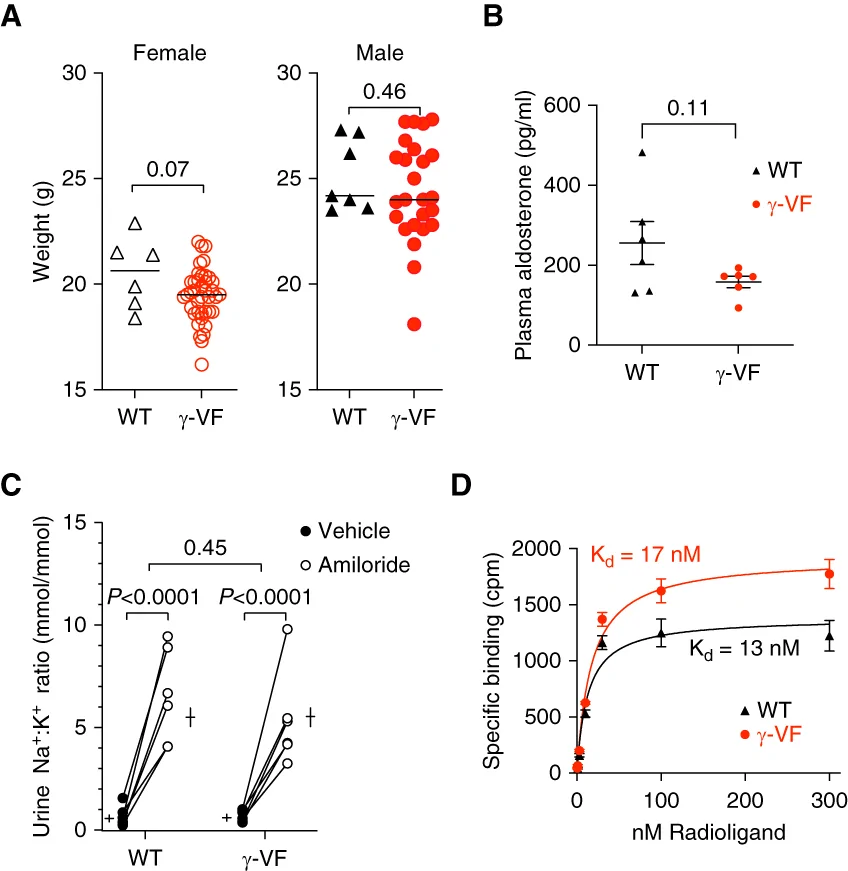
**ENaC*γ*-VF mouse line is physiologically indistinguishable from wild-type at baseline.** (A) Male and female wild-type C57BL/6 and ENaC*γ*-VF mice were weighed at approximately aged 9–11 weeks. Female wild-type mice weighed 20.53±1.18 g (*n*=6), and female ENaC*γ*-VF mice weighed 19.46±1.15 g (*n*=40). Male wild-type mice weighed 25.14±0.96 g (*n*=7), and male ENaC*γ*-VF mice weighed 24.42±2.54 g (*n*=25). Data are shown as mean±SD. (B) Plasma aldosterone levels were measured in male mice only. For each genotype, *n*=6 mice were analyzed. Wild-type mice exhibited a mean aldosterone level of 255.5±53.67, whereas ENaC*γ*-VF mice showed a mean of 158.3±14.46. Data are presented as mean±SEM and were analyzed by a two-tailed *t* test. (C) Amiloride response assay in wild-type and ENaC*γ*-VF mice. The graph shows individual paired responses to vehicle and amiloride for each mouse, with overlaid bars indicating mean±SEM (*n*=6 per group). A two-way ANOVA revealed a significant effect of amiloride treatment compared with vehicle, but no significant difference between genotypes, indicating that the tagged allele supports normal ENaC function. (D) Saturation binding of [^3^H]benzamil to kidney membrane suspension from wild-type and ENaCγ-VF mice. Increasing concentrations of [^3^H]benzamil (0.5–300 nM) were incubated with kidney membranes for 2 hours minimum at 22°C. Specific binding was determined by subtracting nonspecific binding measured in the presence of 100 *µ*M unlabeled phenamil. Data represent one experiment performed in triplicate; points show mean±SD of triplicate measurements. The curve was fit with a one-site–specific binding model using nonlinear regression (GraphPad Prism) to obtain *K*_*d*_. WT, wild-type; γ-VF, γ subunit - mVenus and FLAG.

Blood electrolyte levels in ENaC*γ*-VF mice were within the normal physiological range (Table 1). Body weight and plasma aldosterone levels were also comparable between the ENaC*γ*-VF and wild-type mice, indicating preserved systemic physiology (Figure 2, A and B and Supplemental Table 1). After amiloride administration, urinary Na^+^:K^+^ ratios increased comparably in ENaC*γ*-VF and wild-type mice (Figure 2C).^52^ Radioligand filter-binding assays using tritiated [^3^H] benzamil detected specific binding in kidney membranes from both genotypes (Figure 2D).^29^ Apparent dissociation constants were similar (*K*_*d*_=13 nM for wild-type and 17 nM for ENaC*γ*-VF), indicating that the C-terminal tag does not alter benzamil affinity. These values are comparable with reported IC_50_ values for benzamil inhibition of ENaC-mediated transport measured by electrophysiology, as well as binding affinities of amiloride analogs in native membrane preparations, supporting that the observed binding reflects a native-like pharmacological profile.^28,53,54^ Consistent with this, immunoblot analysis of ENaC*γ*-VF revealed banding patterns comparable with wild-type, including distinct full-length and cleaved species that are shifted to higher apparent molecular weight in the ENaC*γ*-VF samples, and indicating that the *γ* subunit proteolytic processing is preserved in the ENaC*γ*-VF line (Supplemental Figure 2, A–C).

**Table 1:** Plasma sodium and potassium levels in wild-type and ENaC*γ*-VF mice

Electrolyte	Wild-Type (*n*=3)	ENaC*γ*-VF (*n*=3)
Na^+^	146 (1.7) mmol/L	147 (2.7) mmol/L
K^+^	3.7 (0.4) mmol/L	3.8 (0.5) mmol/L

Values are means (SD) of wild-type C57BL/6 and homozygous ENaC*γ*-VF mice under baseline conditions. ENaC*γ*-VF, epithelial sodium channel γ - mVenus FLAG.

### Detection and Characterization of Native ENaC Complexes Using FSEC and SiMPull

The mVenus tag enables fluorescence-based detection of *γ*-containing ENaC assemblies (Figure 3A). We used FSEC^39^ to analyze detergent-solubilized tissue lysates after ultracentrifugation, with the supernatant used for analysis, thereby capturing the solubilized pool of ENaC from both intracellular and plasma membrane compartments. Under these conditions, the resulting chromatograms display broad and, in some cases, multiple fluorescence peaks, consistent with a heterogeneous population of mVenus-containing species. In lung and kidney lysates, fluorescence is distributed across elution volumes between 11 and 16 ml, consistent with a mixture of *γ*-containing assemblies of varying size, likely including fully assembled channels as well as partially assembled or unassembled subunits (Figure 3B). In addition to these species, fluorescence detected at later elution volumes likely reflects a combination of lower molecular weight mVenus-containing species and background tissue autofluoresence. Importantly, control lysates from wild-type mice, which lack the mVenus tag, nevertheless exhibit a detectable fluorescence signal in the mVenus channel that elutes near the expected position of free mVenus, indicating that at least part of the late-eluting signal derives from endogenous tissue autofluorescence rather than tagged protein (Supplemental Figure 2D). By contrast, colon lysates show markedly reduced signal within the 11–16 ml elution range, consistent with lower basal abundance of *γ*-containing ENaC complexes in this tissue. This observation aligns with the known regulation of *β* and *γ* ENaC subunits by aldosterone in the colon, where expression is low under basal conditions compared with kidney, where *β* and *γ* are constitutively expressed.^30,55^

**Figure 3:**
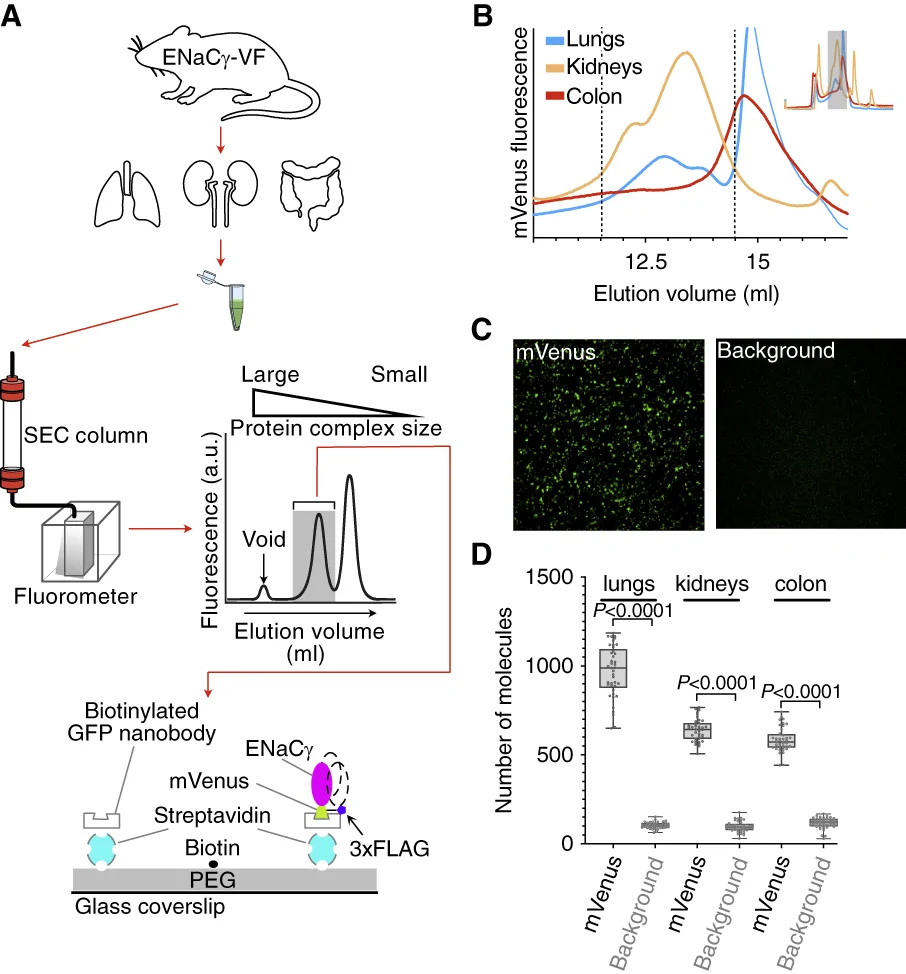
**ENaC*γ*-VF mouse enables subunit-specific detection of native ENaC complexes using FSEC and SiMPull.** (A) Schematic workflow illustrating the experimental pipeline. Tissues (lung, kidney, colon) were harvested from ENaC*γ*-VF mice, homogenized in Tris-based buffer, and followed by solubilization. Cleared supernatants were injected onto a Superose 6 Increase column for FSEC analysis, and fractions corresponding to high-molecular-weight complexes were subsequently analyzed by SiMPull. (B) Representative FSEC traces showing mVenus fluorescence from lung, kidney, and colon lysates. Fluorescent peaks indicate the presence of *γ*-containing ENaC complexes in each tissue. (C) Representative TIRF images of SiMPull experiments from lung lysates. mVenus-tagged ENaC complexes were captured using a biotinylated anti-GFP/mVenus nanobody. A background control image was collected from a flow chamber lacking the nanobody to demonstrate specificity. (D) Quantification of SiMPull signal from lung, kidney, and colon tissues. Each condition represents data pooled from 40 TIRF images per sample, analyzed using the ComDet plugin in ImageJ. Statistical analysis was performed in GraphPad Prism. FSEC, fluorescence-detection size-exclusion chromatography; SEC, size-exclusion chromatography; SiMPull, single-molecule pull-down; TIRF, total internal reflection flourescence microscopy.

To examine ENaC composition at the single-complex level, we performed SiMPull^40^ using a biotinylated anti-GFP/mVenus nanobody^56^ to capture *γ*-containing assemblies for total internal reflection fluorescence (TIRF) imaging. FSEC fractions corresponding to intact ENaC complexes were selected for SiMPull. Robust mVenus signals were detected in lung and kidney samples (Figure 3, C and D). For colon samples, lysates were concentrated before analysis to enable reliable detection. These analyses demonstrate tissue-dependent differences in ENaC abundance.

### Dual-Color FSEC Using a Fluorescent Fab against ENaC*α*

To determine whether *γ*-containing assemblies incorporate ENaC*α*, we developed a fluorescent Fab targeting the *α* subunit. We previously generated monoclonal antibodies again human ENaC subunits, including 7B1 (anti-*α*) and 10D4 (anti-*β*), with 10D4 showing high specificity for human ENaC*β* and 7B1 recognizing both human and mouse ENaC*α* (Figure 4, A and B).^21^ We generated a monovalent fluorescent 7B1 Fab by cloning the variable regions and fusing the heavy chain to mScarlet-I,^45^ yielding 7B1-mScarlet-I (Figure 4C). In recombinant mouse ENaC containing wild-type *α* and *β* subunits and a mVenus-tagged *γ* subunit, 7B1-mScarlet-I coeluted with mVenus in a single peak corresponding to trimeric ENaC (Supplemental Figure 3, A–C), confirming specific binding to *α*-containing complexes. Surface biotinylation experiments, which separate plasma membrane and intracellular fractions, further showed that 7B1-mScarlet-I recognizes *α* in both plasma membrane and intracellular pools (Supplemental Figure 3, D and E).

**Figure 4:**
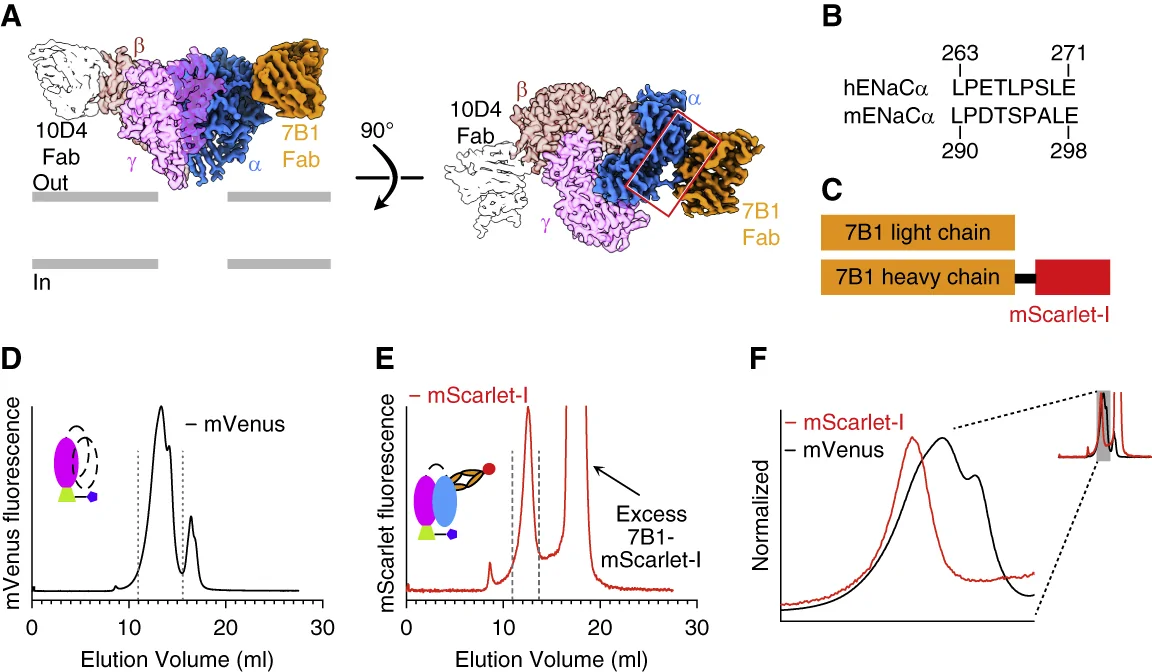
**Dual-subunit detection of native ENaC complexes using 7B1-mScarlet-I and the ENaC*γ*-VF mouse line.** (A) Cryo-EM map of human ENaC in complex with two Fabs, 10D4 and 7B1, shown in side and top-down views. In the top-down view, a red box highlights the interaction site between 7B1 and the *α* subunit. (B) Sequence alignment of the experimentally determined 7B1 epitope region in human and mouse ENaC*α* subunits, revealing high conservation and supporting the potential for cross-reactivity with mouse ENaC. (C) Schematic representation of the 7B1-mScarlet-I Fab construct used to detect the ENaC*α* subunit. The variable regions of the 7B1 heavy and light chains were expressed as a Fab fragment in HEK293S GnTI- cells, with mScarlet-I fused to the C-terminus of the heavy chain. (D) FSEC trace monitoring mVenus fluorescence from lung lysates of ENaC*γ*-VF mice, showing the elution profile of *γ*-containing ENaC complexes. Dotted lines indicate the elution range for mVenus-tagged complexes (11–16 ml). (E) FSEC trace from the same sample after 7B1-mScarlet-I fluorescence, revealing the elution profile of *α*-containing ENaC complexes. Dashed lines show the *α*-associated signal is restricted to a narrower elution range (11–13 ml), suggesting that *α*–*γ* coassemblies represent a more defined subset of the total *γ*-containing complexes. (F) Overlay of the FSEC traces shown in (D) and (E), plotted on the same axes to facilitate direct comparison of elution profiles and peak positions. The inset shows the full normalized traces for each condition. The main panel displays a magnified view of the gray-shaded region indicated in the inset, highlighting the relative alignment of the major peaks. Cryo-EM, cryogenic electron microscopy; Fab, fragment antigen-binding; HEK, human embryonic kidney.

We next examined native *γ*-containing complexes isolated from tissue. For these experiments, detergent solubilized, clarified lysates were used without compartmental separation, thereby capturing ENaC from both intracellular and plasma membrane pools. After FLAG-based enrichment and elution, addition of 7B1-mScarlet-I revealed coelution of mVenus and mScarlet-I fluorescence (Figure 4, D–F), demonstrating incorporation of *α* and *γ* into the same native complexes. Strikingly, mVenus fluorescence spanned a broader elution range (11–16 ml), whereas 7B1-mScarlet-I signal was confined to the 11–13 ml window. These data indicate that *α*-containing complexes represent a defined subset within a broader population of *γ*-containing assemblies, revealing molecular heterogeneity in native ENaC complexes.

### ENaC*γ*-Containing Complexes Associate with Regulatory Proteins *In Vivo*

The restricted *α*-containing peak and broader *γ*-containing distribution suggested that *γ* participates in multiple different assemblies. To define the composition of these assemblies, we performed affinity purification of *γ*-containing complexes from lung, kidney, and colon tissues followed by mass spectrometry (Figure 5A). Although a broader set of proteins was identified, we focus here on previously characterized ENaC-associated and regulatory factors to benchmark the performance of the assay. The complete dataset, specifically proteins that were not detected in wild-type control, is provided in Supplemental Table 2. These experiments were performed on detergent-solubilized, clarified lysates without compartmental fractionation, thereby capturing *γ*-containing complexes from both intracellular and plasma membrane pools. Affinity isolation using the C-terminal FLAG tag and elution recovered ENaC*γ* together with *α* and *β* (Figure 5, B–D and Supplemental Table 2), confirming integrity of assembled channels. In addition to core subunits, regulatory proteins were detected, including the E3 ubiquitin ligase Nedd4-2^57^ and the endoplasmic reticulum chaperone calnexin.^58–60^ Strikingly, the 11*β*-hydroxysteroid dehydrogenase type 2 (annotated as DHI2 in the mass spectrometry dataset) was detected in kidney and colon samples, but not in lung. This distribution is consistent with its established enrichment in aldosterone-responsive epithelia and low expression in lung tissues. The results suggest that *γ*-containing ENaC complexes are recovered within their native tissue-specific regulatory environments. Importantly, these proteins were not detected in parallel purification from wild-type kidney tissues, which lack the 3xFLAG tag (Supplemental Figure 4 and Supplemental Tables 2 and 3), supporting that their recovery is not because of nonspecific binding to the affinity resin. Because solubilization was performed under mild conditions designed to preserve native assemblies, the recovered material likely includes multiprotein complexes. Thus, the identified proteins may reflect a combination of direct interactors and proteins associated within shared cellular or membrane microenvironments. In this context, the detection of known ENaC regulatory factors (*e.g*., Nedd4-2) supports the biological relevance of the purification, while tissue-restricted proteins such as 11*β*-hydroxysteroid dehydrogenase type 2 are consistent with recovery of ENaC within its native epithelial context. However, these data do not distinguish between direct physical interactions and indirect coassociation, and further studies will be required to resolve the nature of these relationships. Together, these findings indicate that *γ*-containing ENaC assemblies can be isolated in association with components of their broader regulatory milieu, encompassing both fully assembled *αβγ* channels and proteins linked to trafficking, quality control, and tissue-specific regulation, consistent with the broader elution profile observed by FSEC.

**Figure 5:**
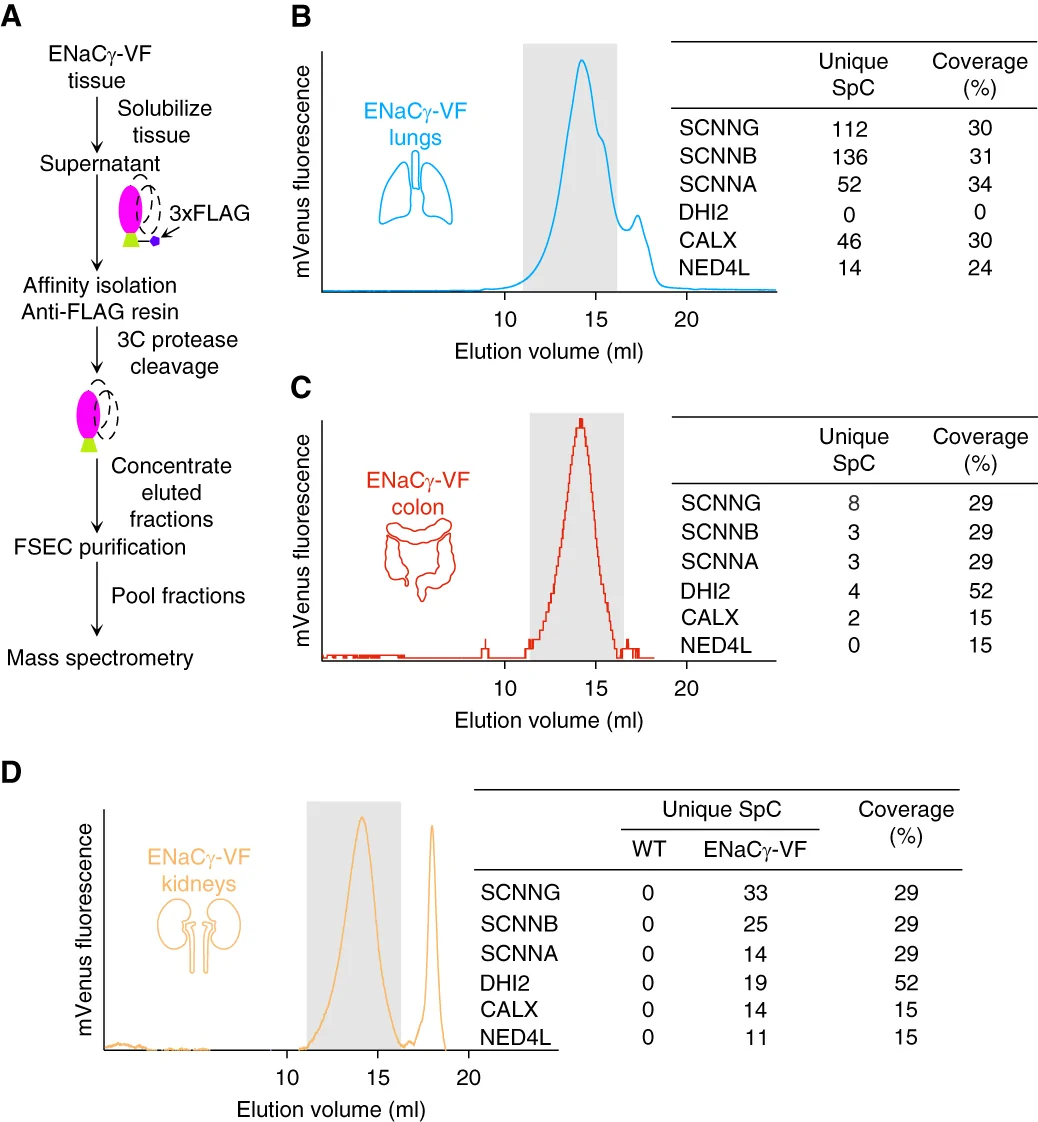
**ENaC complex purification enables tissue-specific interactome analysis by mass spectrometry.** (A) Schematic workflow illustrating the purification pipeline. Tissues (lung, kidney, colon) from ENaC*γ*-VF mice were solubilized under nondenaturing conditions. *γ*-containing ENaC complexes were enriched by affinity capture using the C-terminal FLAG tag, eluted by 3C protease cleavage, and analyzed by mass spectrometry. (B–D) Representative FSEC traces from the lung (B), colon (C), and kidney (D) showing mVenus fluorescence, confirming successful enrichment of *γ*-containing complexes. The gray-shaded region indicates the fractions that were collected, pooled, and used for mass spectrometry analysis. A second prominent peak is observed and corresponds to background fluorescence detected in kidney tissue, independent of ENaC-containing complexes. Mass spectrometry results identifying proteins that copurify with ENaC*γ* from each tissue, with values reflecting unique spectral counts and sequence coverage (%), revealing both shared and tissue-enriched interactors and demonstrating the utility of this approach for defining native ENaC-associated complexes in a tissue-specific context. Protein identifiers shown reflect mass spectrometry annotations (*e.g*., SCNNG, SCNNB, SCNNA, NED4L, CALX, and DHI2), corresponding to ENaC*γ*, ENaC*β*, ENaC*α*, Nedd4-2, calnexin, and 11*β*-HSD2, respectively. 11*β*-HSD2, 11*β*-hydroxysteroid dehydrogenase type 2; CALX, calnexin.

## Discussion

This study establishes the ENaC*γ*-VF knock-in mouse line as a physiologically faithful platform for dissecting the composition, assembly, and regulatory environment of native ENaC complexes across epithelial tissues. Endogenous fluorescent tagging combined with biochemical and single-molecule approaches overcomes longstanding technical barriers imposed by ENaC's low abundance, membrane localization, and complex subunit stoichiometry. Preservation of normal electrolyte handling and pharmacology in homozygous ENaC*γ*-VF mice confirms that the C-terminal mVenus-3C-3xFLAG fusion maintains native channel function, providing confidence that downstream analyses reflect *bona fide* ENaC biology.

The ENaC*γ*-VF allele is broadly compatible with standard biochemical and imaging workflows. The mVenus tag enables fluorescence-based detection by FSEC, confocal imaging, and TIRF microscopy, while the 3xFLAG epitope supports affinity purification and native-state elution using 3C protease. In addition, both mVenus and FLAG facilitate detection by conventional immunoblotting with widely available commercial antibodies, integrating the model into common laboratory assays (Supplemental Figure 2C). These features allow intact ENaC assemblies to be isolated without disrupting subunit composition or regulatory interactions. Thus, the ENaC*γ*-VF mouse provides a practical and adaptable foundation for examining ENaC at molecular, cellular, and tissue scales.

Application of this platform is inherently centered on the *γ* subunit because both isolation and detection strategies enrich for *γ*-containing assemblies. As such, the complexes analyzed here represent a *γ*-defined population and may underrepresent ENaC assemblies that lack *γ*. Within this *γ*-centric view, FSEC reveals a broad distribution of *γ*-containing complexes across the lung, kidney, and colon (Figure 4, D and F), consistent with *γ* participating in multiple assemblies corresponding to distinct stages of channel assembly, maturation, and turnover. Mass spectrometry further supports this view, identifying regulatory factors that copurify with *γ*-containing assemblies, including the E3 ubiquitin ligase Nedd4-2 and the endoplasmic reticulum chaperone calnexin (Figure 5). The recovery of tissue-specific regulatory proteins across organs suggests that ENaC is isolated within its native physiological context, including factors associated with hormone-responsive epithelia. Dual-color FSEC further demonstrates that *α* incorporation is restricted to a narrower subset within this broader *γ* population (Figure 4, E and F), indicating that fully assembled *αβγ* channels represent only a fraction of the total *γ*-containing assemblies. Altogether, these findings support a model in which *γ* is present across multiple assembly intermediates and regulatory contexts under basal conditions.

This interpretation is consistent with prior quantitative studies reporting that *α* subunit abundance is substantially lower than that of *β* and *γ* in native epithelia, suggesting that only a subset of available *γ* participates in fully assembled channels at any given time.^30^ This imbalance was also observed in colon, where *α* is constitutively expressed, while *β* and *γ* expression are regulated by aldosterone, further supporting a model in which *α* availability limits formation of complete ENaC complexes under physiological conditions.

At the level of epithelial transport, ENaC activity is widely recognized as a rate-limiting determinant of sodium absorption across tissues such as the kidney, lung, and colon.^1,61^ Within the channel complex, however, distinct subunits contribute to different limiting steps depending on tissue context and regulatory state.^30,31^ Early biochemical and expression studies identified *α* as essential for channel assembly and surface expression, consistent with a limiting role in formation of functional *αβγ* complexes.^62–65^ By contrast, *β* and *γ* subunits have been more strongly implicated in trafficking, surface abundance, and stability.^66–68^ For example, mutations in the C-termini of *β* or *γ* in Liddle syndrome disrupt Nedd4-2-mediated ubiquitination, increasing channel surface abundance and activity.^69,70^ Similarly, airway-specific overexpression of *β* in transgenic mice drives sodium hyperabsorption, airway surface dehydration, impaired mucociliary clearance, mucus obstruction, and chronic lung inflammation, producing a well-established model of CF-like muco-obstructive lung disease that highlights the sensitivity of ENaC function to subunit dosage.^68,71,72^ Together, these examples illustrate how changes in subunit abundance or stability can drive pathological ENaC phenotypes.

Within the scope of the present experiments enabled by this new mouse line, our data support a model in which ENaC assembly and function are shaped by the relative availability and regulation of individual subunits. In contrast to transgenic models that perturb subunit expression, the ENaC*γ*-VF line preserves endogenous regulatory balance, allowing direct interrogation of ENaC architecture without inducing pathological remodeling. These observations indicate that distinct subunits constrain ENaC at different stages of assembly and regulation, with *α* availability limiting formation of fully assembled channels and *β*/*γ* influencing trafficking and stability in a tissue-dependent manner.

More broadly, the ENaC*γ*-VF platform enables a transition from indirect inference to direct molecular definition of native ENaC assemblies, providing an entry point for systematically dissecting ENaC composition and regulation under physiological conditions. By enabling isolation, visualization, and quantification of intact ENaC complexes under nondenaturing conditions, this system establishes a foundation for linking subunit composition and regulatory state to tissue-specific ENaC function. Because complementary tools continue to emerge for detecting additional ENaC subunits, particularly *β*, and for structural analysis of native complexes, the ENaC*γ*-VF model will serve as a foundational resource for defining the molecular principles that govern ENaC regulation across epithelial systems. In future work, this platform can be applied to examine how disease states remodel ENaC assembly and regulatory interactions, linking molecular architecture to disordered Na^+^ handling in conditions such as salt-sensitive hypertension, cystic fibrosis, and pseudohypoaldosteronism.

## Supplementary Material

## Data Availability

Original data generated for the study will be made available upon reasonable request to the corresponding author. Data Type: Image Data, Statistical Analysis Plan, Mass spec data, and fluorescence-based data. Reason for Restricted Access: All data described in this manuscript, as well as the ENaCγ-VF mouse line, are available from the corresponding author upon reasonable request.

## Declarative Statements

All animal experiments were conducted in accordance with the NIH Guide for the Care and Use of Laboratory Animals or an equivalent standard that meets or exceeds the ethical and welfare requirements outlined in the NIH Guide. All protocols were approved by the appropriate institutional animal care and use committee. This research was posted on a preprint server. DOI: 10.64898/2026.01.23.701393.

## Supplemental Material

Supplemental Table 1. Body weights of ENaC*γ*-VF and WT mice (9–11 week old).

Supplemental Table 2. Mass spectrometry analysis of purified ENaC complexes from colon, kidney, and lung tissues.

Supplemental Table 3. Mass spectrometry results from the wild-type control sample.

Supplemental Figure 1. Immunofluorescence staining of kidney tissue from heterozygous ENaC*γ*-VF mice.

Supplemental Figure 2. Workflow and validation of purification from mouse tissues.

Supplemental Figure 3. Validation of 7B1-mScarlet-I as a tool for detecting mouse ENaC*α*.

Supplemental Figure 4. Control purification workflow using wild-type mice to assess nonspecific binding.
