# Supplementary material for: Differential Assembly of Native Epithelial Sodium Channel Complexes Across Mouse Epithelial Tissues
Source: Kidney360. 2026 Jun 15;7(9):1985–97. doi: 10.34067/KID.0000001261 (PMC13641531; doi:10.34067/KID.0000001261)
Supplement: Supplementary file 2 [file kidney360-7-1985-s002.pdf]

Differential Assembly of Native ENaC Complexes Across Mouse Epithelial Tissues

Arpita Bharadwaj[1], Joshua Curry[2], Xiao-Tong Su[2], Romina Barria Maturana[1], James A. McCormick[2,3], David H. Ellison[2,3], and Isabelle Baconguis[1,*]

[1]Vollum Institute, Oregon Health & Science University, Portland, Oregon 97239

[2]Division of Nephrology and Hypertension, Department of Medicine, Oregon Health & Science University, Portland, Oregon 97239

[3]Department of Chemical Physiology and Biochemistry, Oregon Health & Science University, Portland, Oregon 97239

[*]Correspondence: Dr. Isabelle Baconguis, bacongui@ohsu.edu

Table of Contents

Supplemental Table 1	3
Supplemental Table 2	4
Supplemental Table 3	7
Supplemental Figure 1	13
Supplemental Figure 2	15
Supplemental Figure 3	18
Supplemental Figure 4	20

Group	Mean weight (g)	n	p-value (vs WT)
Wild-type females	20.53 ± 1.18	6	
ENaCγ-VF females	19.46 ± 1.15	40	**0.07**
Wild-type males	25.14 ± 0.96	7	
ENaCγ-VF males	24.42 ± 2.54	25	**0.46**

Supplemental Table 1. Body weights of ENaCγ-VF and WT mice (9-11 weeks old).

Values are mean ± SD in grams (g). Unpaired two-tailed t-tests, n as indicated.

Accession	Coverage (%)	Unique SpC			Coverage (%)	Unique SpC
		Wild type kidney	**ENaCγ-VF colon**	**ENaCγ-VF kidney**		**ENaCγ-VF lung**
**sp|Q9WU39|SCNNG_MOUSE**	**29**	**0**	**8**	**33**	**30**	**112**
**sp|Q9WU38|SCNNB_MOUSE**	**29**	**0**	**3**	**25**	**31**	**136**
sp|P51661|DHI2_MOUSE	52	0	4	19	0	0
**sp|Q61180|SCNNA_MOUSE**	**29**	**0**	**3**	**14**	**34**	**52**
sp|P35564|CALX_MOUSE	15	0	2	14	30	46
sp|Q8CFI0|NED4L_MOUSE	15	0	0	11	24	14
sp|Q8BXZ1|TMX3_MOUSE	17	0	0	7	0	0
sp|Q9EP69|SAC1_MOUSE	14	0	0	7	19	2
sp|Q6ZQI3|MLEC_MOUSE	19	0	0	6	24	7
sp|P97363|SPTC2_MOUSE	15	0	0	6	0	0
sp|O54734|OST48_MOUSE	17	0	0	5	17	10
sp|Q9D024|CCD47_MOUSE	12	0	0	5	24	4
sp|Q80TN4|DJC16_MOUSE	9	0	0	5	0	0
sp|Q8BHN3|GANAB_MOUSE	7	0	0	5	5	3
sp|Q8C7X2|EMC1_MOUSE	5	0	0	5	8	3
sp|Q3V3R1|C1TM_MOUSE	7	0	2	4	0	0
sp|Q9D1D4|TMEDA_MOUSE	26	0	0	4	32	10
sp|P47740|AL3A2_MOUSE	10	0	0	4	12	5
sp|Q3TDQ1|STT3B_MOUSE	7	0	0	4	12	9
sp|P21271|MYO5B_MOUSE	2	0	0	4	0	0
sp|P56395|CYB5_MOUSE	34	0	0	3	0	0
sp|O08547|SC22B_MOUSE	21	0	0	3	0	0
sp|Q8BLF1|NCEH1_MOUSE	12	0	0	3	16	2
sp|Q8VBZ3|CLPT1_MOUSE	10	0	0	3	16	11
sp|Q8BJY1|PSMD5_MOUSE	9	0	0	3	0	0
sp|Q8BU33|HACL2_MOUSE	8	0	0	3	0	0
sp|Q8R0X7|SGPL1_MOUSE	7	0	0	3	0	0
sp|P70414|NAC1_MOUSE	6	0	0	3	0	0
sp|Q9R049|AMFR_MOUSE	5	0	0	3	0	0
sp|P59158|S12A3_MOUSE	4	0	0	3	0	0
sp|Q3U1J4|DDB1_MOUSE	3	0	0	3	14	8
sp|P07356|ANXA2_MOUSE	11	0	2	2	23	6
sp|Q6P5E4|UGGG1_MOUSE	2	0	1	2	5	5
sp|O55022|PGRC1_MOUSE	26	0	0	2	38	9
sp|Q80UU9|PGRC2_MOUSE	20	0	0	2	37	7

sp|Q9CQB5|CISD2_MOUSE	16	0	0	2	0	0
sp|Q8R5J9|PRAF3_MOUSE	16	0	0	2	0	0
sp|Q9CQU3|RER1_MOUSE	15	0	0	2	15	5
sp|Q8BXV2|BRI3B_MOUSE	13	0	0	2	17	4
sp|Q60866|PTER_MOUSE	12	0	0	2	0	0
sp|P47758|SRPRB_MOUSE	11	0	0	2	0	0
sp|P35278|RAB5C_MOUSE	11	0	0	2	0	0
sp|Q8C0L0|TMX4_MOUSE	8	0	0	2	0	0
sp|O35704|SPTC1_MOUSE	7	0	0	2	17	2
sp|Q3TDN2|FAF2_MOUSE	7	0	0	2	23	7
sp|Q8CAQ8|MIC60_MOUSE	7	0	0	2	0	0
sp|O70252|HMOX2_MOUSE	7	0	0	2	0	0
sp|P01901|HA1B_MOUSE	7	0	0	2	0	0
sp|Q9DBX6|CP2S1_MOUSE	6	0	0	2	12	3
sp|O09173|HGD_MOUSE	6	0	0	2	0	0
sp|P62334|PRS10_MOUSE	6	0	0	2	0	0
sp|Q9CQY5|MAGT1_MOUSE	6	0	0	2	9	3
sp|Q9QYI4|DJB12_MOUSE	6	0	0	2	0	0
tr|Q3UNV2|Q3UNV2_MOUSE	5	0	0	2	0	0
sp|Q9QZE5|COPG1_MOUSE	5	0	0	2	7	3
sp|Q99J23|GHDC_MOUSE	4	0	0	2	21	3
sp|Q8BSY0|ASPH_MOUSE	4	0	0	2	14	5
sp|P46978|STT3A_MOUSE	4	0	0	2	0	0
sp|O08795|GLU2B_MOUSE	3	0	0	2	9	2
sp|O35082|KLOT_MOUSE	3	0	0	2	0	0
sp|Q6GQT9|NOMO1_MOUSE	2	0	0	2	0	0
sp|P14873|MAP1B_MOUSE	1	0	0	2	0	0
sp|Q7TMY8|HUWE1_MOUSE	1	0	0	2	0	0
sp|Q3UNX5|ACSM3_MOUSE	38	0	9	1	0	0
sp|Q99K01|PDXD1_MOUSE	6	0	3	1	0	0
sp|O88685|PRS6A_MOUSE	13	0	0	1	0	0
sp|P61161|ARP2_MOUSE	9	0	0	1	0	0
sp|Q99JY9|ARP3_MOUSE	7	0	0	1	0	0
sp|A2A5R2|BIG2_MOUSE	3	0	0	1	0	0
sp|Q9ESE1|LRBA_MOUSE	1	0	0	1	0	0

Supplemental Table 2. Mass spectrometry analysis of purified ENaC complexes from colon, kidney, and lung tissues.

γ-containing ENaC complexes were affinity-purified and further clarified by size-exclusion chromatography from detergent-solubilized lysates of ENaCγ-VF mouse tissues and analyzed by mass spectrometry. Wild-type control tissues were processed in parallel using the exact same purification workflow, including identical solubilization, affinity enrichment, size-exclusion chromatography conditions, and collection of the same fraction from the same elution volume, to ensure that isolation conditions were directly comparable. Proteins listed represent those detected in ENaCγ-VF samples but not in parallel purifications from wild-type control purification (spectral counts = 0 in wild-type), thereby enriching for proteins specifically associated with the tagged complexes. For colon and kidney samples, which were processed and analyzed within the same mass spectrometry run, peptide spectral counts are shown for each tissue, and sequence coverage (%) reflects the combined dataset from both samples. Proteins are ranked based on spectral counts observed in the kidney samples, from highest to lowest. Lung samples were processed and analyzed in a separate mass spectrometry run; therefore, spectral counts and sequence coverage (%) for lung are reported independently and are not directly comparable to those of colon and kidney. Spectral counts provide a relative measure of protein detection within each sample but do not represent absolute abundance or stoichiometry.

Accession	Coverage (%)	Unique SpC
sp|E9Q3L2|PI4KA_MOUSE	39	54
sp|Q8CIG8|ANM5_MOUSE	55	52
sp|Q99J09|MEP50_MOUSE	72	46
sp|Q8K010|OPLA_MOUSE	42	41
tr|F6R7E8|F6R7E8_MOUSE (+1)	12	33
sp|Q8VDN2|AT1A1_MOUSE_family	37	30
sp|P56480|ATPB_MOUSE	65	30
sp|Q01853|TERA_MOUSE	59	25
sp|P52332|JAK1_MOUSE	31	20
sp|Q9JJ28|FLII_MOUSE	28	20
sp|Q64331|MYO6_MOUSE	27	17
sp|Q62468|VILI_MOUSE	40	17
sp|Q91ZA3|PCCA_MOUSE	37	17
tr|Q792Z1|Q792Z1_MOUSE	19	17
sp|O35488|S27A2_MOUSE	31	16
sp|Q05920|PYC_MOUSE	22	16
sp|Q03265|ATPA_MOUSE	39	15
sp|E9Q6P5|TTC7B_MOUSE	33	14
sp|Q99MR8|MCCA_MOUSE	37	14
sp|Q8BMS1|ECHA_MOUSE	26	14
sp|Q8CFE4|SCYL2_MOUSE	24	12
sp|P63038|CH60_MOUSE	56	12
sp|A2ARV4|LRP2_MOUSE	6	11
sp|Q8R0Y6|AL1L1_MOUSE	26	11
sp|P47738|ALDH2_MOUSE	40	11
sp|Q99MN9|PCCB_MOUSE	42	11
sp|Q8BGB2|TTC7A_MOUSE	28	11
sp|Q810B6|ANFY1_MOUSE	28	11
sp|Q60604|SCIN_MOUSE	33	11
sp|O88338|CAD16_MOUSE	23	11
sp|Q3ULD5|MCCB_MOUSE	37	10
sp|P50516|VATA_MOUSE	27	10
sp|Q9JHJ0|TMOD3_MOUSE	44	9
sp|Q9CZ13|QCR1_MOUSE	26	9
sp|Q61838|PZP_MOUSE	14	9
sp|P16460|ASSY_MOUSE	44	8
sp|P13020|GELS_MOUSE	19	8

sp|Q505D1|ANR28_MOUSE	14	8
sp|Q9DB77|QCR2_MOUSE	27	7
sp|P28825|MEP1A_MOUSE	14	7
sp|Q3V1L4|5NTC_MOUSE	20	7
sp|P54071|IDHP_MOUSE	20	7
sp|Q3UZ39|LRRF1_MOUSE	20	7
tr|A8DUK4|A8DUK4_MOUSE	63	7
sp|Q8K2B3|SDHA_MOUSE	24	6
sp|P57780|ACTN4_MOUSE	14	6
sp|P16858|G3P_MOUSE	42	6
sp|P14094|AT1B1_MOUSE	25	6
sp|P80314|TCPB_MOUSE	19	6
sp|O88844|IDHC_MOUSE	23	6
sp|P97449|AMPN_MOUSE	14	5
sp|Q9DCX2|ATP5H_MOUSE	57	5
sp|Q9DBR0|AKAP8_MOUSE	27	5
sp|P70398|USP9X_MOUSE	6	5
sp|Q8C729|HYCC2_MOUSE	16	5
sp|P01942|HBA_MOUSE	37	5
sp|P11983|TCPA_MOUSE	13	5
sp|Q9WV92|E41L3_MOUSE	14	4
sp|P41216|ACSL1_MOUSE	10	4
sp|Q8BFR5|EFTU_MOUSE	18	4
sp|Q8CIE6|COPA_MOUSE	9	4
sp|Q91V92|ACLY_MOUSE	6	4
sp|Q62433|NDRG1_MOUSE	24	4
sp|Q9Z2I9|SUCB1_MOUSE	15	4
sp|Q9QUM9|PSA6_MOUSE	21	4
sp|P07724|ALBU_MOUSE	12	4
sp|P80317|TCPZ_MOUSE	18	4
sp|Q9DBG6|RPN2_MOUSE	28	3
sp|P36993|PPM1B_MOUSE	30	3
sp|Q8CC88|VWA8_MOUSE	11	3
sp|Q9DBM2|ECHP_MOUSE	14	3
sp|P50544|ACADV_MOUSE	13	3
sp|P10126|EF1A1_MOUSE	12	3
sp|Q91WK0|LRRF2_MOUSE	19	3
sp|Q8CCF0|PRP31_MOUSE	17	3
sp|Q6P9N1|HYCCI_MOUSE	14	3

sp|P42932|TCPQ_MOUSE	11	3
sp|P80318|TCPG_MOUSE	11	3
sp|P97742|CPT1A_MOUSE	9	3
sp|Q60605|MYL6_MOUSE	33	3
sp|Q61189|ICLN_MOUSE	31	3
sp|Q8BG32|PSD11_MOUSE	22	3
sp|Q9DB20|ATPO_MOUSE	21	3
sp|P00405|COX2_MOUSE	20	3
sp|Q9D0M3|CY1_MOUSE	20	3
sp|Q8BVE3|VATH_MOUSE	14	3
sp|Q91VD9|NDUS1_MOUSE	11	3
sp|Q9EQ20|MMSA_MOUSE	10	3
sp|P53395|ODB2_MOUSE	9	3
sp|P19096|FAS_MOUSE	3	3
sp|Q9CQQ7|AT5F1_MOUSE	18	3
sp|Q9D2G2|ODO2_MOUSE	9	3
sp|Q9DCT2|NDUS3_MOUSE	21	3
sp|Q91Y97|ALDOB_MOUSE	13	3
sp|Q9CZ04|CSN7A_MOUSE	13	3
sp|P14685|PSMD3_MOUSE	11	3
sp|Q8VEK3|HNRPU_MOUSE	6	3
sp|Q9DCW4|ETFB_MOUSE	19	3
sp|P46471|PRS7_MOUSE	9	3
sp|P01872|IGHM_MOUSE	9	3
sp|P99026|PSB4_MOUSE	12	3
sp|O55125|NIPS1_MOUSE	6	3
sp|E9Q557|DESP_MOUSE	6	2
sp|P09055|ITB1_MOUSE	13	2
sp|Q9NYQ2|HAOX2_MOUSE	11	2
sp|Q9CQA3|SDHB_MOUSE	28	2
sp|Q9Z2I8|SUCB2_MOUSE	10	2
sp|Q91VR2|ATPG_MOUSE	7	2
sp|P26039|TLN1_MOUSE	3	2
sp|P47754|CAZA2_MOUSE	21	2
sp|Q60930|VDAC2_MOUSE	14	2
sp|P15105|GLNA_MOUSE	13	2
sp|Q9Z2V5|HDAC6_MOUSE	8	2
sp|O88343|S4A4_MOUSE	5	2
sp|P0CG49|UBB_MOUSE (+4)	33	2

sp|P35550|FBRL_MOUSE	19	2
sp|P62806|H4_MOUSE	18	2
sp|P56135|ATPK_MOUSE	26	2
sp|Q9R1P0|PSA4_MOUSE	10	2
sp|Q68FD5|CLH1_MOUSE	4	2
sp|P62908|RS3_MOUSE	16	2
tr|Q91VA7|Q91VA7_MOUSE	14	2
sp|Q61767|3BHS4_MOUSE (+1)	12	2
sp|P51881|ADT2_MOUSE	11	2
sp|Q8K274|KT3K_MOUSE	10	2
sp|Q9Z2U0|PSA7_MOUSE	10	2
sp|Q99JY0|ECHB_MOUSE	8	2
sp|Q9CR68|UCRI_MOUSE	8	2
sp|Q9QYB5|ADDG_MOUSE	7	2
sp|Q8R1B4|EIF3C_MOUSE	7	2
sp|Q9DCL9|PUR6_MOUSE	7	2
sp|Q922D8|C1TC_MOUSE	6	2
sp|P62702|RS4X_MOUSE	16	2
sp|P38647|GRP75_MOUSE	26	2
sp|P97450|ATP5J_MOUSE	26	2
sp|Q9R1P4|PSA1_MOUSE	19	2
sp|Q7TNG8|LDHD_MOUSE	18	2
sp|P49722|PSA2_MOUSE	17	2
sp|P62852|RS25_MOUSE	14	2
sp|Q8BH95|ECHM_MOUSE	11	2
sp|P57746|VATD_MOUSE	11	2
sp|Q8VC30|TKFC_MOUSE	9	2
sp|Q920Q8|NS1BP_MOUSE	7	2
sp|Q923I7|SC5A2_MOUSE	5	2
sp|Q99K67|AASS_MOUSE	5	2
sp|Q9D687|S6A19_MOUSE	4	2
sp|P80316|TCPE_MOUSE	4	2
sp|Q99JI4|PSMD6_MOUSE	9	2
sp|O88544|CSN4_MOUSE	7	2
sp|Q922B2|SYDC_MOUSE	6	2
sp|Q07417|ACADS_MOUSE	6	2
sp|Q91WV7|SLC31_MOUSE	4	2
sp|Q8JZZ0|UD3A2_MOUSE	4	2
sp|Q60597|ODO1_MOUSE	2	2

sp|O55143|AT2A2_MOUSE	17	1
sp|Q91YQ5|RPN1_MOUSE	22	1
sp|Q8BWT1|THIM_MOUSE	21	1
sp|Q80XN0|BDH_MOUSE	20	1
sp|Q9R112|SQOR_MOUSE	21	1
sp|O70325|GPX4_MOUSE	24	1
tr|A0A571BF69|A0A571BF69_MOUSE	3	1
sp|Q02257|PLAK_MOUSE	13	1
sp|P62835|RAP1A_MOUSE (+1)	18	1
sp|Q9QYC0|ADDA_MOUSE	12	1
sp|Q922D4|PP6R3_MOUSE	6	1
sp|Q920R6|VPP4_MOUSE	5	1
sp|Q8VDM4|PSMD2_MOUSE	4	1
sp|Q8BTM8|FLNA_MOUSE	5	1
sp|P61979|HNRPK_MOUSE	12	1
sp|P62821|RAB1A_MOUSE	19	1
sp|P50518|VATE1_MOUSE	10	1
sp|P24270|CATA_MOUSE	8	1
sp|P99028|QCR6_MOUSE	37	1
sp|P56391|CX6B1_MOUSE	29	1
sp|P17751|TPIS_MOUSE	23	1
sp|P19783|COX41_MOUSE	18	1
sp|P99029|PRDX5_MOUSE	16	1
sp|Q9WVJ2|PSD13_MOUSE	15	1
sp|O09061|PSB1_MOUSE	14	1
sp|Q9D8W5|PSD12_MOUSE	13	1
sp|Q9R1P1|PSB3_MOUSE	11	1
sp|Q9DCH4|EIF3F_MOUSE	10	1
sp|P55096|ABCD3_MOUSE	7	1
sp|P80313|TCPH_MOUSE	7	1
sp|Q8VCH0|THIKB_MOUSE (+1)	5	1
sp|Q76MZ3|2AAA_MOUSE	5	1
sp|Q8BMF4|ODP2_MOUSE	5	1
sp|Q62470|ITA3_MOUSE	4	1
sp|Q5SYD0|MYO1D_MOUSE	3	1
sp|Q9ES97|RTN3_MOUSE	2	1
sp|Q7TSI3|PP6R1_MOUSE	2	1
sp|Q9Z2B5|E2AK3_MOUSE	2	1
sp|P01869|IGH1M_MOUSE	14	1

sp|Q9CQ13|COPRS_MOUSE	29	1
sp|O70435|PSA3_MOUSE	9	1
sp|Q9WUA3|PFKAP_MOUSE	5	1
sp|P23116|EIF3A_MOUSE	2	1
sp|P62315|SMD1_MOUSE	20	1
sp|Q99LF4|RTCB_MOUSE	6	1

Supplemental Table 3. Mass spectrometry results from the wild-type control sample. This table lists proteins identified in the wild-type control sample and includes only proteins with detectable spectral counts.

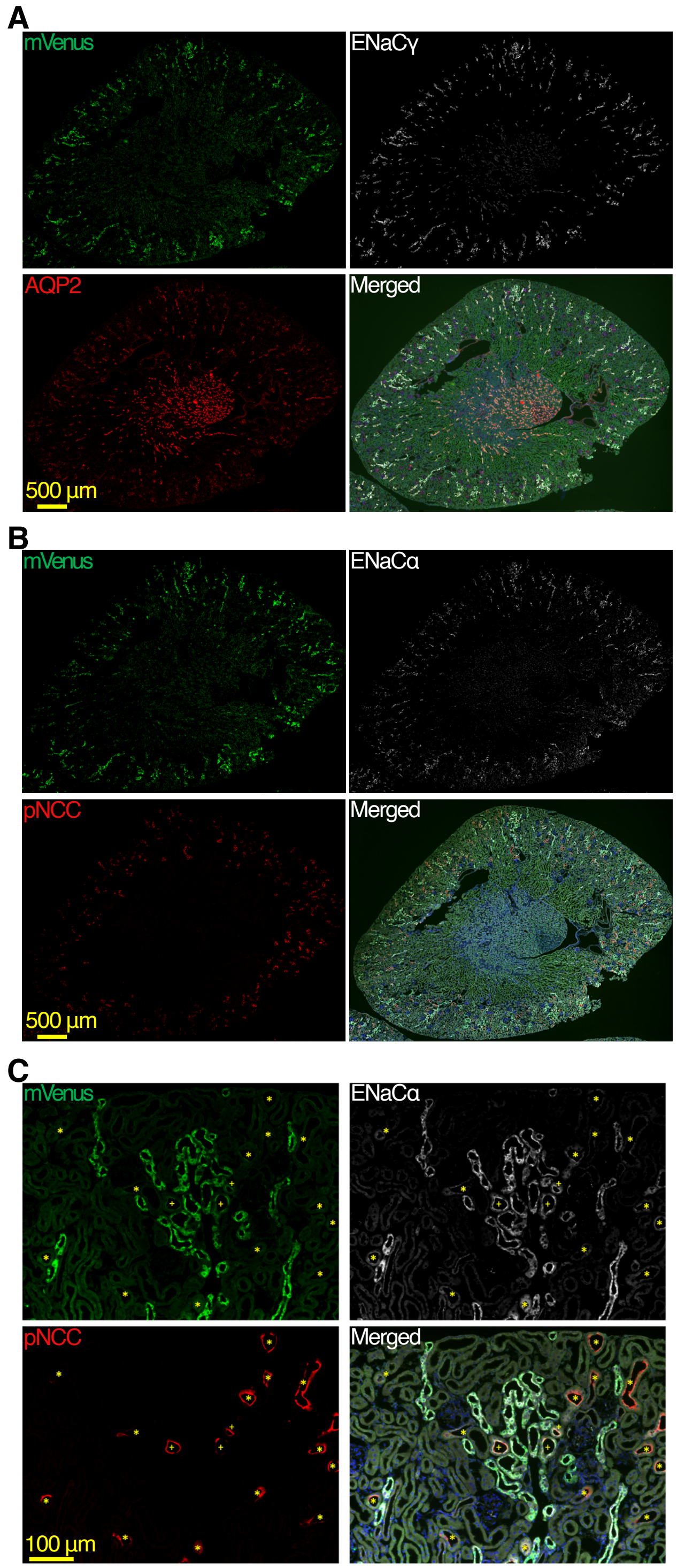

Supplementary Figure 1

Supplemental Figure 1. Immunofluorescence staining of kidney tissue from heterozygous ENaCγ-VF mice.

A. Representative immunofluorescence image showing co-localization of mVenus, ENaCγ, and aquaporin-2 in kidney tissue. mVenus signal was detected using a FITC-conjugated goat anti-GFP primary antibody (Rockland, 1:100) and Alexa Fluor 488 donkey anti-goat secondary antibody (1:500). Endogenous ENaCγ was detected using a rabbit anti-rat ENaCγ primary antibody (StressMarq, 1:500) targeting the C-terminus, and Alexa Fluor 647 donkey anti-rabbit secondary antibody (1:500). Aquaporin-2 (AQP2) was stained using a mouse anti-AQP2 primary antibody (Santa Cruz, 1:50) and Alexa Fluor 555 donkey anti-mouse secondary antibody (1:500). **B**. Representative immunofluorescence image showing co-localization of mVenus, ENaCα, and pNCC in kidney tissue. mVenus signal was detected using a FITC-conjugated anti-GFP primary antibody (goat, Rockland, 1:100) and Alexa Fluor 488 donkey anti-goat secondary antibody (1:500). Endogenous ENaCα was detected using a rabbit anti-ENaCα primary antibody (generous gift of J. Loffing[41]) targeting the C-terminus, and Alexa Fluor 647 donkey anti-rabbit secondary antibody. The phosphorylated form of the Na^{+}-Cl^{-} cotransporter (NCC) was stained using a chicken anti-NCC (generously provided by J. Wade[42]) and Cy3 donkey anti-chicken secondary antibody. **C**. Close-up view highlighting localization of tagged mVenus, ENaCα, and pNCC. * indicate DCT1 (pNCC+/ENaCα-); + indicate DCT2 (pNCC+/ENaCα+).

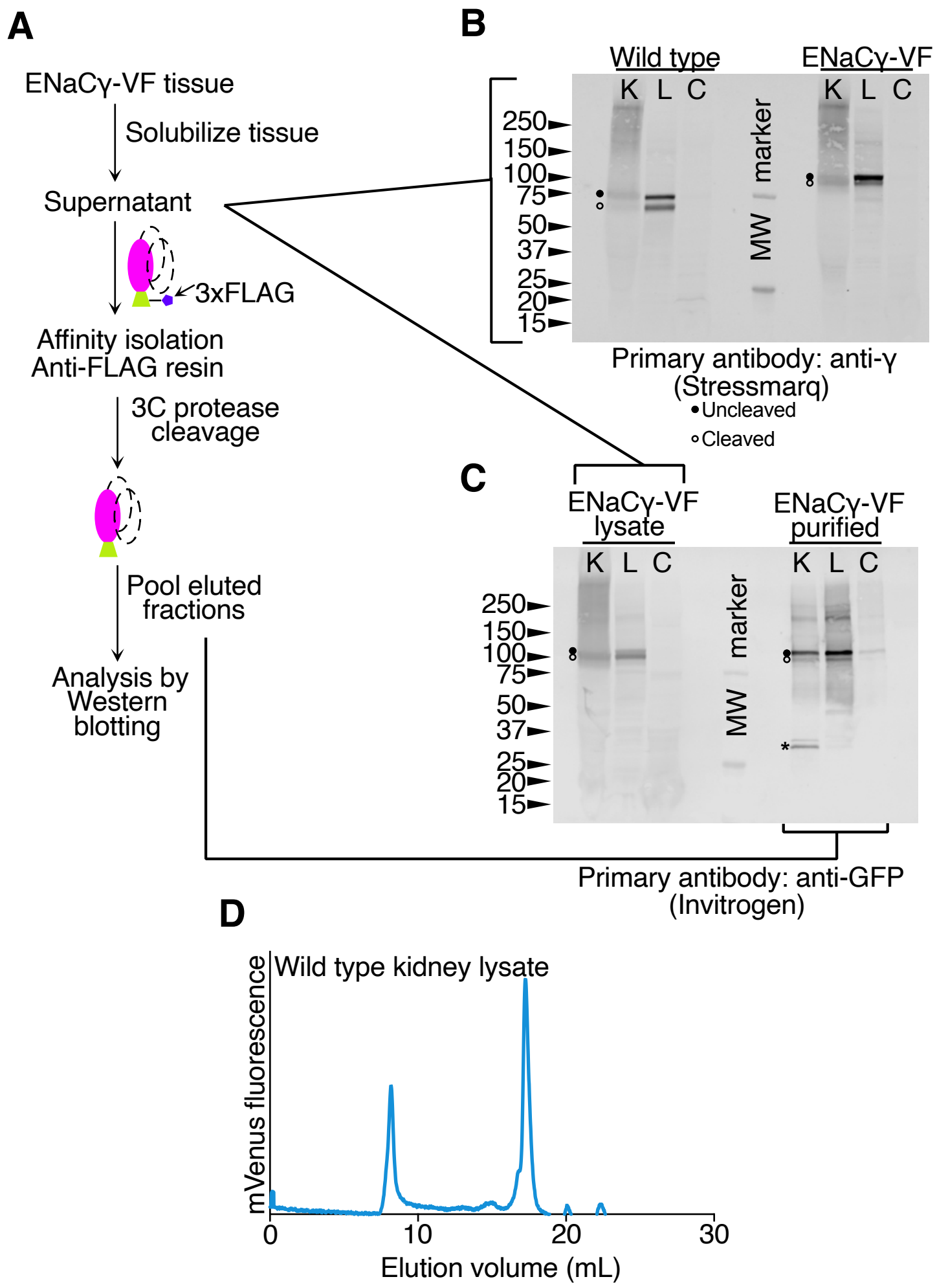

Supplementary Figure 2

Supplemental Figure 2. Workflow and validation of purification from mouse tissues. **A**. Schematic of the sample preparation workflow used for purification and downstream analysis. **B**. Western blot analysis of kidney (K), lung (L), and colon (C) lysates from wild-type and ENaCγ-VF mice probed for the ENaCγ subunit (Rabbit anti-rat ENaCγ primary antibody (Stressmarq, 1:1000) and IRDye 800 CW Goat anti-rabbit secondary antibody (LICORbio, 1:20,000). Distinct bands corresponding to full-length (filled circle) and proteolytically cleaved (open circle) ENaCγ are observed in both genotypes. In ENaCγ-VF mice, these bands are shifted to higher apparent molecular weight, consistent with the addition of the mVenus-3C-3xFLAG fusion (~31 kDa). The expected molecular weights are ~74.6 kDa (WT full-length) vs ~105.8 kDa (γ-VF full-length), and ~53.4 kDa (WT cleaved) vs ~84.6 kDa (γ-VF cleaved), assuming cleavage at the canonical furin recognition site in ENaCγ. The separation between full-length and cleaved γ-VF species is less apparent in crude lysates due to reduced resolution at higher molecular weight. **C**. Western blot analysis of lysates and purified material from kidney (K), lung (L), and colon (C) tissues from ENaCγ-VF mice probed with anti-GFP primary antibody (Invitrogen, 1:4000) and IRDye 800 CW Goat anti-rabbit secondary antibody (LICORbio, 1:20,000) to detect the mVenus tag fused to ENaCγ. The same lysate samples shown in panel b were re-run alongside purified material on the same gel. In lysates, separation between full-length and cleaved γ-VF species remains limited, whereas the purified material shows improved resolution, allowing clearer distinction of these species. This enhanced separation upon enrichment supports the assignment of full-length and cleaved ENaCγ-VF and enables detection in colon tissue under baseline conditions, where signal is not readily detectable in whole lysates. An additional lower-molecular-weight band (asterisk;

between 25-37 kDa molecular weight marker) is detected by the GFP antibody specifically in the purified samples but not in lysates. This species may arise from partial proteolysis during the purification procedure, which includes an overnight binding step at 4 °C. Interestingly, this band is consistent with a fluorescent specie observed by FSEC that elutes between ~15-20 mL in the mVenus channel shown in Figure 5d, supporting that it contains the tagged fragment. This peak is observed in purified kidney samples but not in lung or colon by either Western blot or FSEC (Figure 5B-D). **D**. Representative FSEC trace of wild-type kidney tissue showing an autofluorescent peak that elutes near the expected free mVenus elution volume.

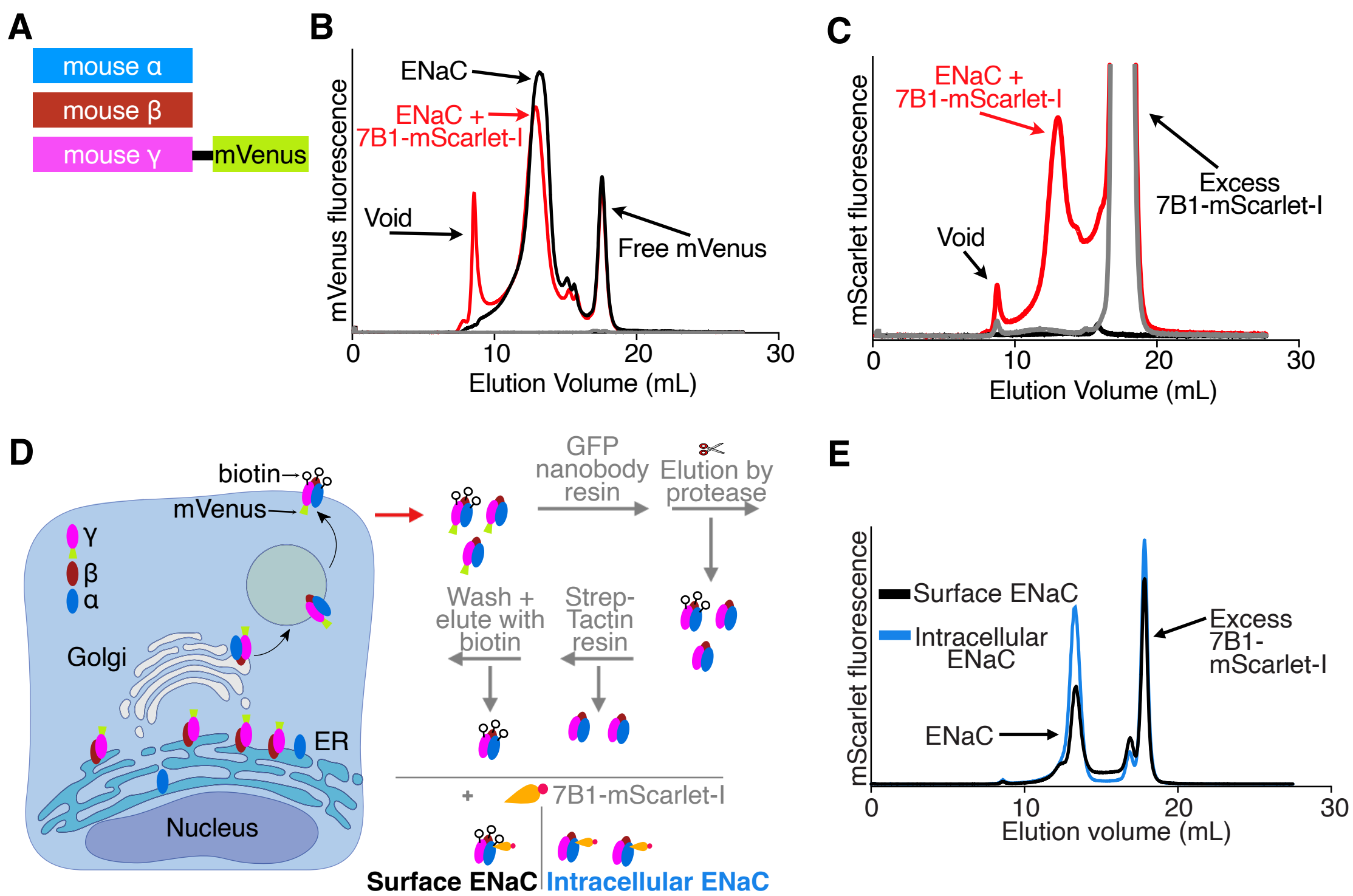

Supplementary Figure 3

Supplemental Figure 3. Validation of 7B1-mScarlet-I as a tool for detecting mouse ENaCα.

A. Schematic of the recombinant mouse ENaC subunit constructs used for validation. The α and β subunits were expressed with wild-type sequences, while the γ subunit was C-terminally fused to mVenus, mirroring the configuration in ENaCγ-VF mice. **B, C.** FSEC traces showing mVenus (b) and mScarlet-I fluorescence (c) from lysates of cells expressing recombinant mouse ENaC. Co-elution of the two signals confirms that 7B1-mScarlet-I binds specifically to complexes containing ENaCα, validating its use in detecting α subunits within γ-containing assemblies. **D.** Schematic illustration of ENaC complexes located inside the cell and in the plasma membrane. Only surface ENaC is exposed to membrane-impermeable biotin. **E.** FSEC traces of purified surface-expressed ENaC protein (black trace) and intracellular ENaC pool (blue trace) monitored on the mScarlet-I channel fused to the 7B1 Fab. The difference in peak height is due to preexisting differences in relative abundance and not a difference in 7B1 recognition.

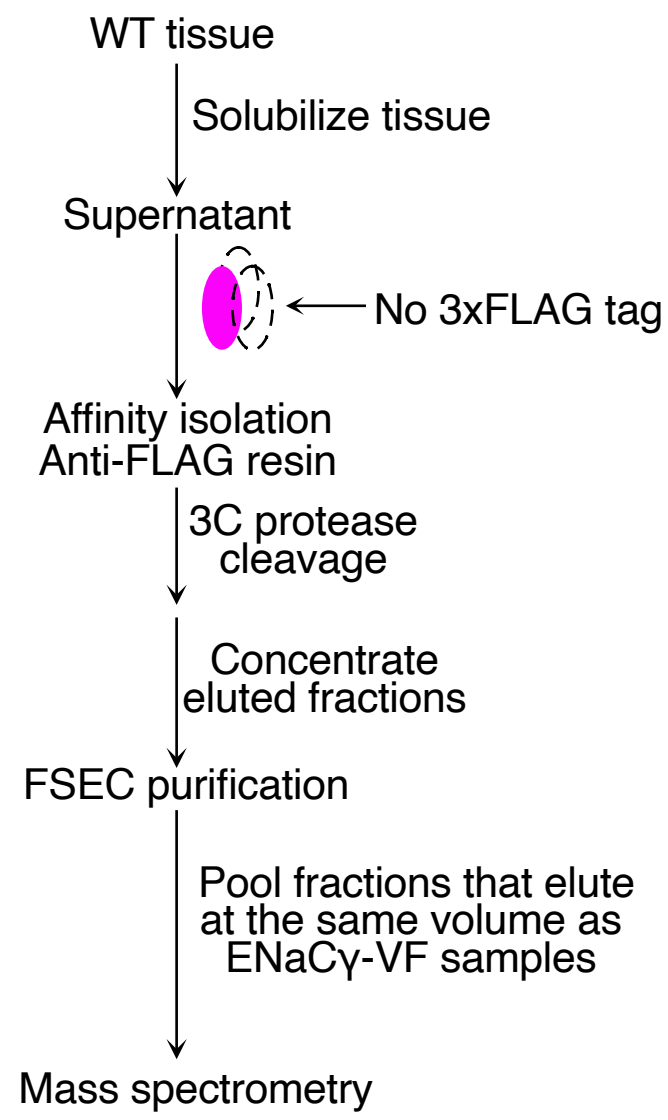

Supplementary Figure 4

Supplemental Figure 4. Control purification workflow using wild-type mice to assess non-specific binding.

Schematic of the purification workflow applied to wild-type mouse tissues, performed in parallel with the ENaCγ-VF workflow. Although the protocol is identical, including tissue solubilization, anti-FLAG affinity capture, and protease elution, wild-type mice lack the C-terminal 3xFLAG tag on ENaCγ and therefore cannot be captured by the anti-FLAG resin. This control serves to identify proteins that may non-specifically bind to the affinity resin and distinguishes true ENaC-associated interactors from background contaminants in the ENaCγ-VF purification experiments.
